# Microbiota-mediated phytate metabolism activates HDAC3 to contribute intestinal homeostasis

**DOI:** 10.1038/s41392-020-00321-5

**Published:** 2020-09-21

**Authors:** Xiaoxing Li, Harry Cheuk Hay Lau, Jun Yu

**Affiliations:** 1 Institute of Precision Medicine, First Affiliated Hospital, Sun Yat-Sen University, Guangzhou, China; 2 Institute of Digestive Disease, Department of Medicine and Therapeutics, State Key Laboratory of Digestive Disease, Li Ka Shing Institute of Health Sciences, CUHK Shenzhen Research Institute, The Chinese University of Hong Kong, Sha Tin, NewTerritories Hong Kong

**Keywords:** Gastroenterology, Microbiology

In a recent study in *Nature*, Wu et al. discovered a fundamental host–microbe interaction that is crucially involved in responding to dietary change thereby maintaining healthy intestinal dynamics.^1^ They demonstrated that HDAC3 activity can be promoted by microbiota-mediated phytate metabolism to contribute intestinal homeostasis and repair.

Intestinal epithelial cells (IECs) form a barrier that limits interactions between the host and gut microbiota to maintain homeostasis.^2^ To facilitate their broad spectrum of functions, IECs are regularly replenished, which is mediated by multiple intrinsic regulators, particularly histone deacetylase (HDAC).^3^ It is now clear that HDAC activation is largely influenced by the commensal microbes and their metabolites, as exemplified by the inhibitory effect of microbiota-derived butyrate on HDAC activity.^4^ Meanwhile, to date, there is inadequate study to assess whether microbial signals can stimulate HDAC-induced epigenetic regulation. In a recent study of Wu et al., they identified a novel microbiota-mediated metabolic pathway that could activate HDAC to contribute intestinal homeostasis.^1^

Butyrate is well-documented for suppressing HDAC activation.^4^ Indeed, colonising germ-free (GF) mice with butyrate-producing bacteria (*Faecalibacterium prausnitzii*) largely inhibited HDAC enzymatic activity in IECs. Wu et al., therefore, expected that HDAC activity would be decreased further in conventionally housed (CNV) mice with replete microbiota, due to the higher level of intestinal butyrate. Yet, this was not the case—IECs from CNV mice exhibited significantly greater HDAC activity, thus suggesting that more complicated regulation rather than butyrate-mediated inhibition alone was involved in the interaction between microbiota and HDAC activity.

Among all members of HDACs, HDAC3 is specifically known for its close relationship with microbiota, as it can integrate microbial signals with responses of IECs to maintain intestinal homeostasis.^3^ Wu and colleagues therefore continued their investigation by knocking out HDAC3 from the IECs of CNV mice (CNV-HDAC3^ΔIEC^). When comparing with wild-type controls, these HDAC3-depleted mice displayed half-fold decrease in HDAC activity, thus confirming that microbiota could promote HDAC3 activation in normal intestinal homeostasis.

Next, Wu and colleagues deciphered the underlying mechanisms and interestingly found that neither common bacterial stimuli (e.g. lipopolysaccharides and flagellin) nor the canonical bacteria-sensing TLR4/MyD88 pathway showed any association with microbiota-mediated HDAC3 activation. The authors then compared the transcriptomes of GF mice-derived IECs with CNV mice-derived IECs, and identified the significant enrichment of genes involved in inositol phosphate metabolism and its signalling molecule inositol trisphosphate (IP3). After exposure to IP3, HDAC3 activity was greatly enhanced in primary IECs and intestinal organoids, of which IP3 supplementation could even counteract the butyrate-mediated inhibition on HDAC3 activity in IECs. Hence, these in vitro data implicated the intricate relationship between IP3 and butyrate against HDAC3 activation.

But how microbiota impacts inositol phosphate metabolism to activate HDAC? In this metabolic pathway dietary phytate (IP6; enriched in nuts and grains) is enzymatic degraded by phytase to produce IP3 or other mono- to penta-phosphorylated inositols (IP1-IP5). Wu and colleagues therefore delivered phytate to intestinal explants of microbiota-replete CNV mice, microbiota-depleted GF mice, or CNV-HDAC3^ΔIEC^ mice. As expected, increased HDAC activity was only observed in IECs isolated from CNV mice after phytate treatment. Of note, commensal *Escherichia coli* is responsible for releasing phytase.^5^ When colonising GF mice with *E. coli*, HDAC activation was significantly boosted in IECs. Wu et al. further validated the in vivo effects of microbiota-derived phytase on HDAC by supplementing CNV mice with either wild-type or phytase-deficient *E. coli*. IP3 enrichment and enhanced IEC-intrinsic HDAC activity were exhibited in mice with phytase-proficient wild-type *E. coli*, whereas these features were abolished in mice with phytase-deficient *E. coli*. Altogether, these data provided convincing evidence on the interaction between microbiota and HDAC activity, which was dependent on phytate metabolism and expression of commensal phytase.

One of the critical functions of IECs is to promote repair upon intestinal damage. However, such recovery is often dampened in patients with ulcerative colitis (UC). Remarkably, Wu et al. identified significant reduction of bacterial phytase in UC patients, thus suggesting that decreased phytate metabolism might contribute to impaired epithelial recovery. The authors then treated GF and CNV mice with dextran sodium sulfate (DSS, which is highly toxic to IECs) with or without phytate addition. The survival of DSS-treated CNV mice with phytate supplementation was greatly ameliorated, while phytate had no impact on the survival of DSS-treated GF mice. After DSS removal, phytate-treated mice exhibited decreased inflammation and phenotypic features of robust epithelial regeneration. Given that tissue repair is regulated by intestinal epithelial stem cells (IESCs), Wu and colleagues further isolated EpCAM^+^ (a protein attributes to cell adhesion, proliferation, and differentiation) IESCs from mice. Under this in vitro setting, IESCs derived from phytate-treated mice showed much greater proliferation than IESCs from vehicle-treated mice. Hence, all these data illustrated the necessity of an intact microbiota in phytate-dependent recovery following intestinal damage by inducing epithelial proliferation.

By contrast, IP3 administration was sufficient to improve discovery from DSS in microbiota-depleted mice, indicating that IP3 might directly integrate with intestinal epithelium to promote repair independent from microbiota. In line with in vivo phenotypes, intestinal organoids from GF mice displayed enhanced proliferation under exposure to IP3. Notably, Wu et al. succeeded to replicate the plausible in vivo antagonising effect between IP3 and butyrate on IECs by utilising in vitro cultures, of which IP3 supplementation could rescue the inhibited growth of butyrate-treated organoids derived from GF mice. The authors further treated colonic organoids from wide-type (GF-HDAC3^FF^) or HDAC3-knockout GF mice (GF-HDAC3^ΔIEC^) with IP3. Consistently, IP3 treatment promoted the growth of GF-HDAC3^FF^-derived organoids, while impaired basal growth and insensitivity to IP3 were observed in GF-HDAC3^ΔIEC^-derived organoids.

**Fig. 1:**
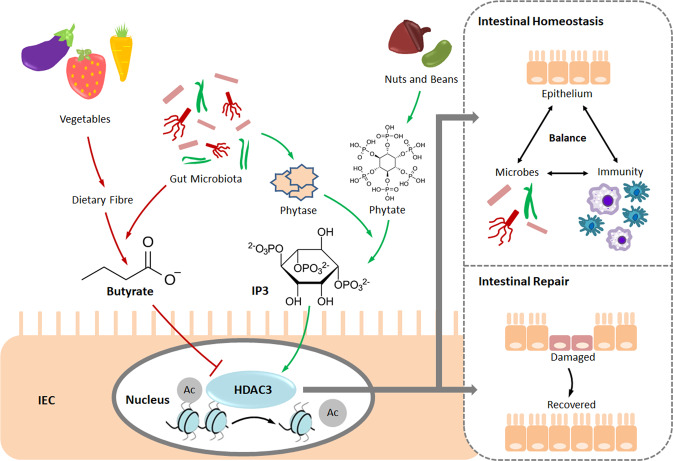
Gut microbes metabolise dietary fibres to produce butyrate, which is known for suppressing the activity of an epigenetic regulator HDAC3. By contrast, metabolising dietary phytate (enriched in nuts and beans) with aid of microbes-secreted phytase can produce IP3, which can induce HDAC3 activation as newly discovered by Wu et al.^1^ HDAC3 promotes the removal of the acetyl group (abbreviated as Ac) from the histone tail of chromatin. While HDAC3 activation can contribute intestinal homeostasis and repair. Hence, HDAC3 acts as an essential regulator that can calibrate intestinal dynamics in response to dietary change

Collectively, Wu et al. identified a novel microbiota-mediated regulatory pathway—commensal phytase degraded dietary phytate to produce IP3, which could directly activate HDAC3 to promote epithelial repair (Fig. 1). Of note, although not being completely elucidated, it is rational to say that intestinal homeostasis is fine-tuned by HDAC3, of which its activity is constantly shifted by the local concentrations of stimulatory phytate-derived IP3 and inhibitory fibre-derived butyrate. In summary, this paper supplements a new pivotal role to the established HDAC3-mediated microbial sensation that contributes epigenetic regulation of healthy intestinal dynamics in response to dietary change.
